# Supplementary material for: Arsenic Contamination of Groundwater Is Determined by Complex Interactions between Various Chemical and Biological Processes
Source: Toxics. 2024 Jan 19;12(1):0. doi: 10.3390/toxics12010089 (PMC11154318; doi:10.3390/toxics12010089)
Supplement: Supplementary file 1 [file toxics-12-00089-s001.zip › toxics-2701881-supplementary.pdf]

toxics

MDPI

Supplementary Materials: Arsenic Contamination of Groundwater is Determined by Complex Interactions between Various Chemical and Biological Processes

Zahid Hassan and Hans V. Westerhoff

S1. Supplementary Figures

In this section figures are presented that are supplemental to the main text.

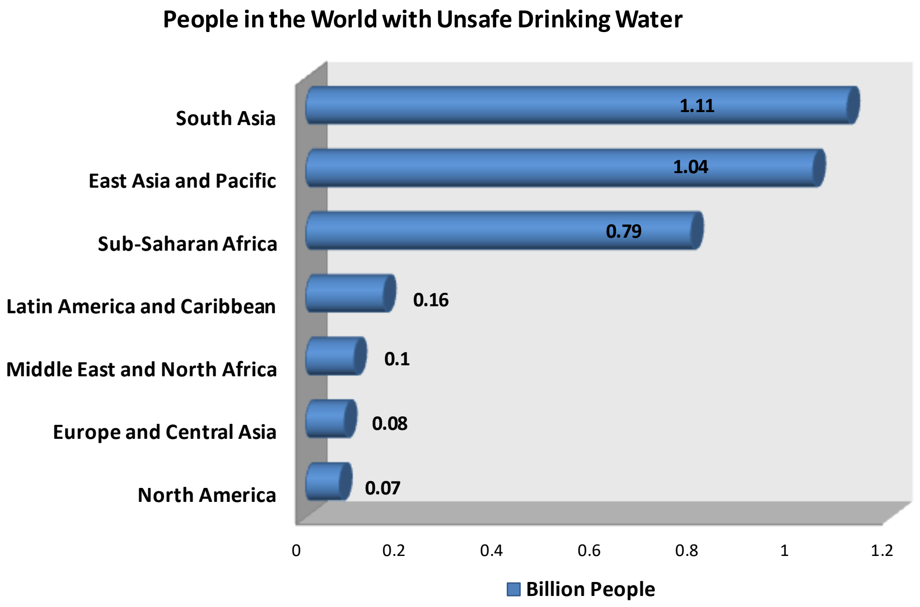

Figure S1. Estimated numbers of people (in billion) in various parts of the world with unsafe drinking water (online data were obtained from WHO/UNICEF as of 2020 [293]).

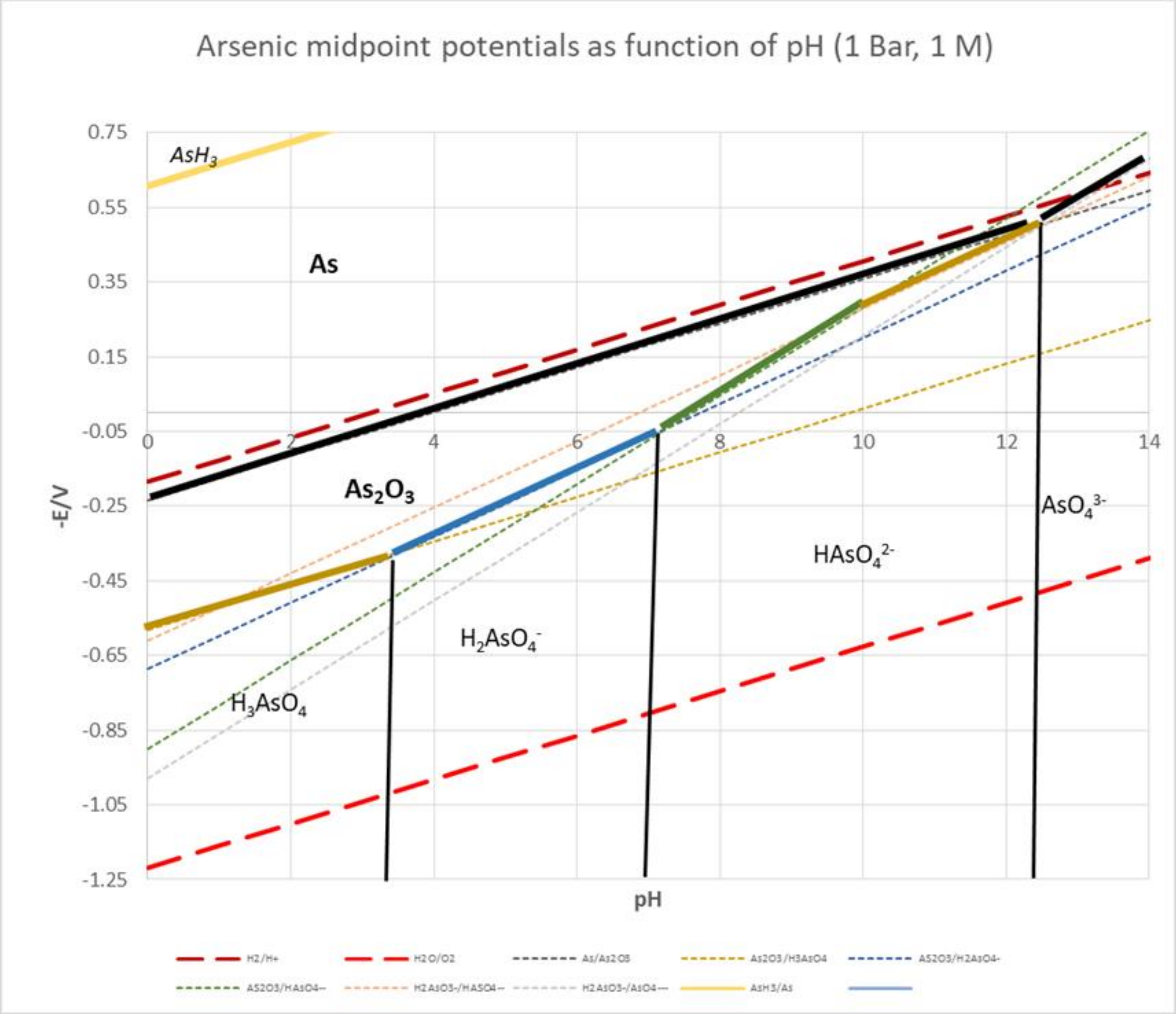

Figure S2. Negative redox potential (='electron potential') versus acidity (-*E*/*pH*) diagram ("mirrored Pourbaix diagram") indicating the thermodynamically lowest Gibbs energy form of arsenic at 25 °C and 101.3 kPa total pressure (=1 bar = 1 atmosphere) at physical chemical standard conditions (1 M, 1 bar). Solid and gaseous species are in bold face and italics, respectively; solutes in roman type. -*E*/V: the negative redox potential in Volt. With *E* (the redox potential as equilibrated with an AgCl/Ag electrode defined in [294]; in the microbiological literature sometimes referred to as *Eh*) we refer to the ambient redox potential. The names indicate the species that is most abundant of all arsenic species in the corresponding area of the plot delineated by the thick lines. The thick colored lines separate the arsenite (As(III)) on the left from the arsenate (As(V)) more to the right; the arsenite occurs as insoluble As_2O_3 under these 1 M standard conditions, making the arsenite essentially immobile in contrast to the arsenous acid (H_3AsO_3) of Fig. 2. At the low -*E* (electron potential) values encountered in equilibrated oxygenated waters, arsenic acid species (i.e., the 'arsenates' H_3AsO_4, H_2AsO^-, $HAsO_4^{2-}$ or AsO_4^{3-}, depending on pH, are more stable than arsenite. At higher -*E* values characteristic of (mildly) reducing conditions As_2O_3 become more stable. The slopes of the lines correspond to the number of protons versus electrons involved in the transition between the two adjacent species; vertical lines correspond to *pKa*'s. The long-dashed red lines enclose the relevant region for aqueous media, i.e., where water is stable versus H_2 and O_2, respectively. The lower red-dashed line indicates the apparent (pH dependent) midpoint potential of the oxygen-water couple, whilst the upper long-dashed red line represents the hydrogen-proton apparent (at 1 mbar partial pressure of hydrogen gas) midpoint potential. Below the former the electron potential –*E* is low enough for water to be oxidized to oxygen gas and above the latter the water protons are reduced to hydrogen gas; only in between these two lines water can be in a stable equilibrium with the ambient electron potential (minus redox potential) -*E*. Thin lines are apparent [55] midpoint potentials used to construct the thick lines. Part of the figure corresponds to a figure in [58].

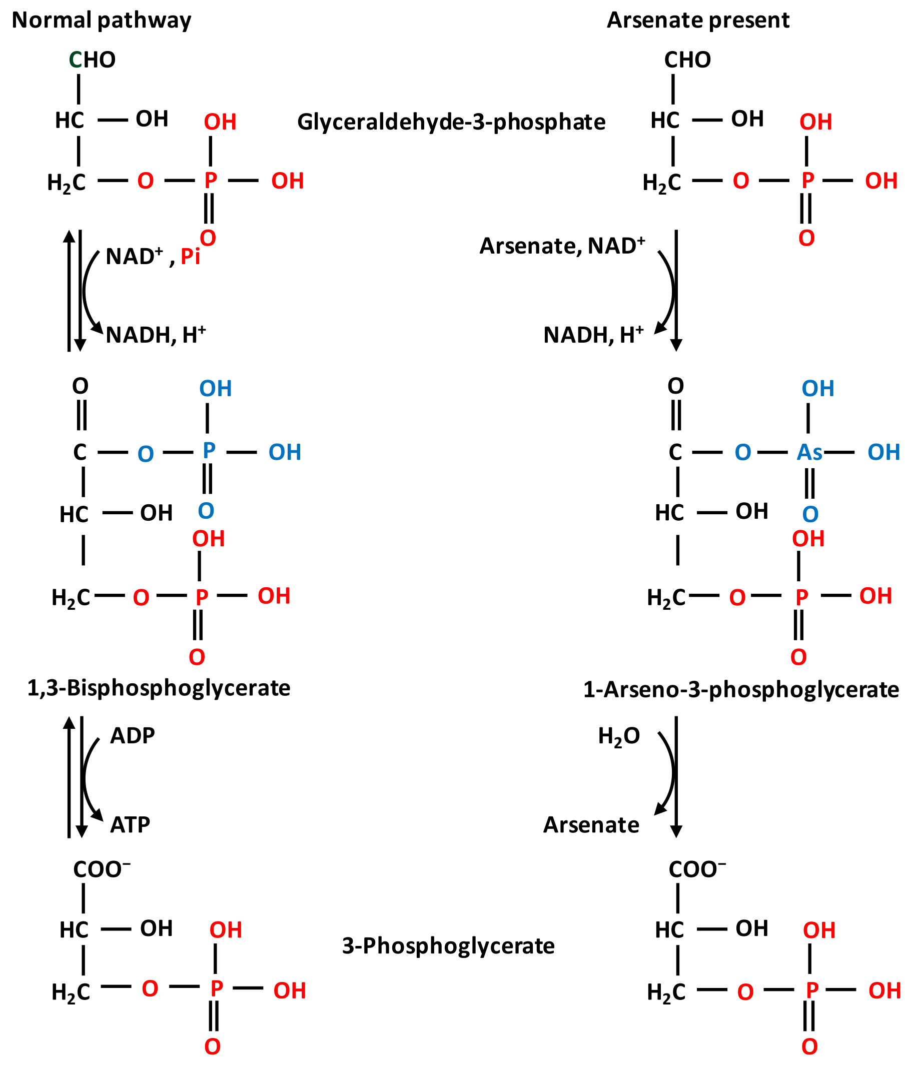

Figure S3. Uncoupling of substrate-level phosphorylation in glycolysis by arsenate [see also [295]]. The normal pathway is shown on the left; the one in the presence of arsenate on the right. In the latter, arsenate can replace inorganic phosphate in glyceraldehyde-3-phosphate dehydrogenase to produce 1-arseno-3-phosphoglycerate instead of 1,3-bisphosphoglycerate. This 1-arseno-3-phosphoglycerate hydrolyzes spontaneously. This yields the next intermediate in the pathway, 3-phosphoglycerate without generating the usual ATP. The term "uncoupling" implies that the pathway proceeds without ATP synthesis whilst the product of the hydrolysis, 3-phosphoglycerate, is the normal glycolytic intermediate.

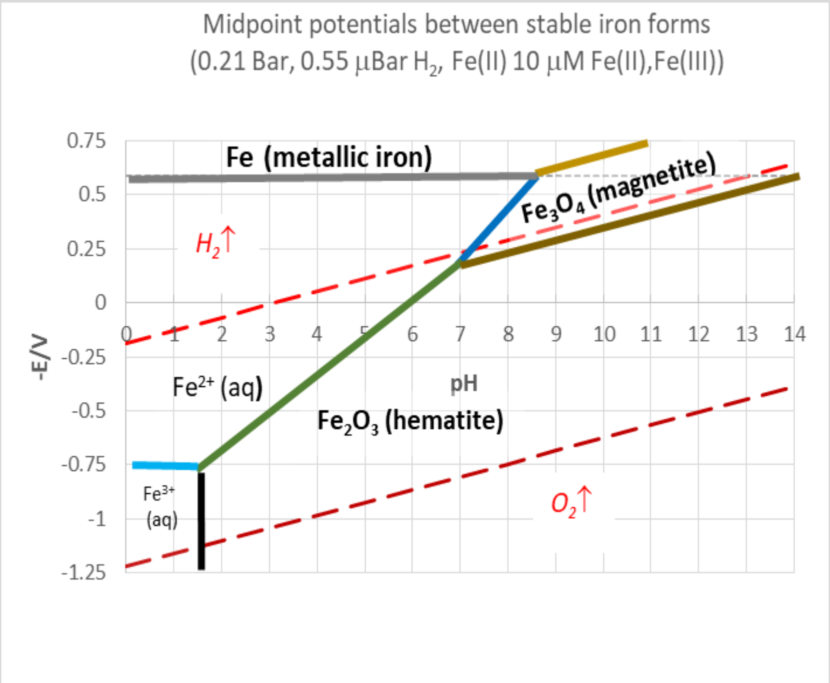

Figure S4. Electron potential (negative redox potential –*E*) versus acidity (-*E* versus pH) diagram. The diagram indicates the thermodynamically lowest Gibbs energy form of iron at 25 °C and 101.3 kPa total pressure (1 atmosphere) at relevant standard conditions (21% O_2, 0.55 μBar H_2, 10 μM ferrous and ferric iron. Solid species are in bold face, others are aqueous solutes. This diagram is analogous to but different from the Pourbaix diagram for iron in [69]. The colored thick lines demarcate the transitions between the various redox states of iron and the vertical black line the pKa of the transition between aqueous ferric iron and the solid hematite. The dashed red lines are the borders beyond which protons form hydrogen gas and water forms molecular oxygen. At ambient and acidic pH and intermediate redox potential, ferrous iron is mostly stable in the aqueous state Fe^{2+}(aq), whilst ferric iron largely occurs at more alkaline pH and higher redox potentials and then in the form of hematite (Fe_2O_3(s) or the only slightly less stable (+0.2 kJ/mol Fe), but more organized and hydrated goethite ($2FeO_2H$(s)=$Fe_2O_3.H_2O$(s)). The disordered, hydrated form of hematite is called ferrihydrite with formula $Fe_2O_3.0.5H_2O$ (s) and is less stable than either other form. At pH > 7 and an electron potential (negative redox potential) -*E*> 0.3V a form with a redox state intermediate between ferrous and ferric is the most stable. It is called magnetite Fe_3O_4(s) ($Fe(III)_2Fe(II)O_4$(s)) as it can be magnetic [69]. Metallic iron is unstable in water (i.e. at –E, pH between the dashed red lines), as it will oxidize to ferrous iron at acidic pH and hematite at neutral and alkaline pH. At neutral pH and ambient redox potentials, iron will thereby be immobile, but it will be mobilized by transitions to acidic pH.

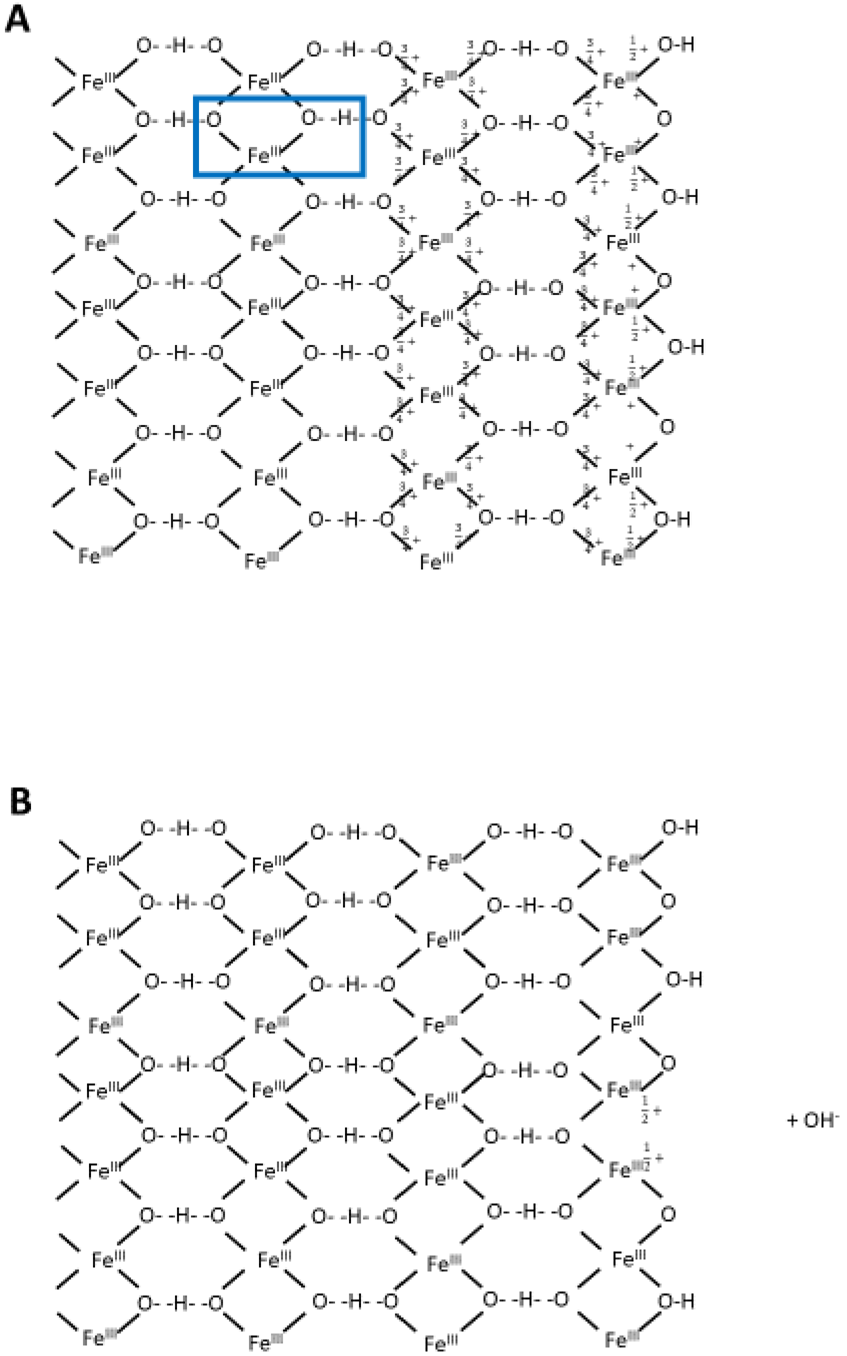
A
B
+ OH-

C

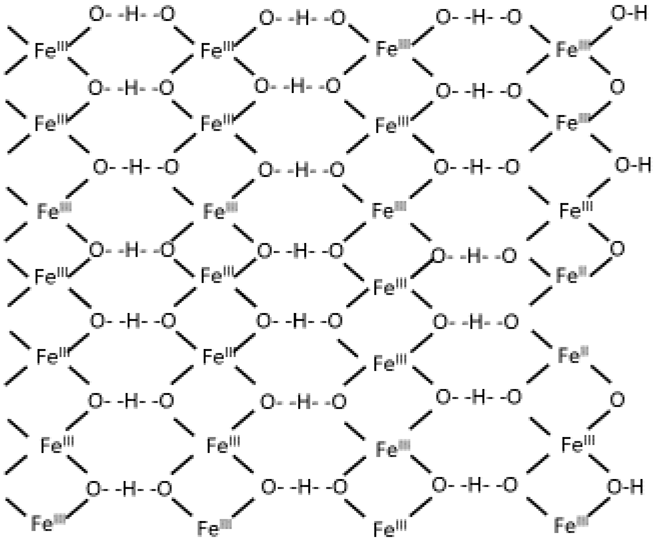

D

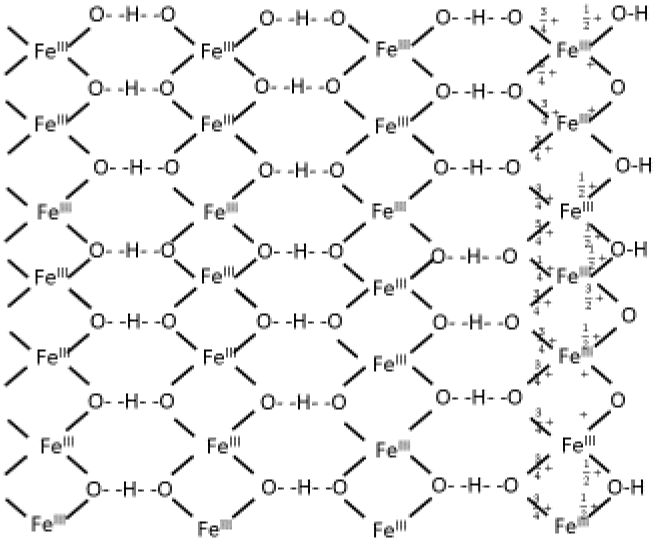

E

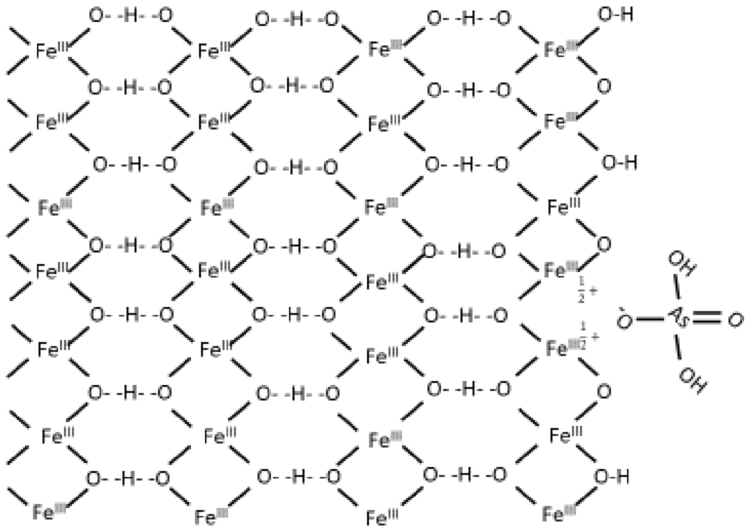

q81

F

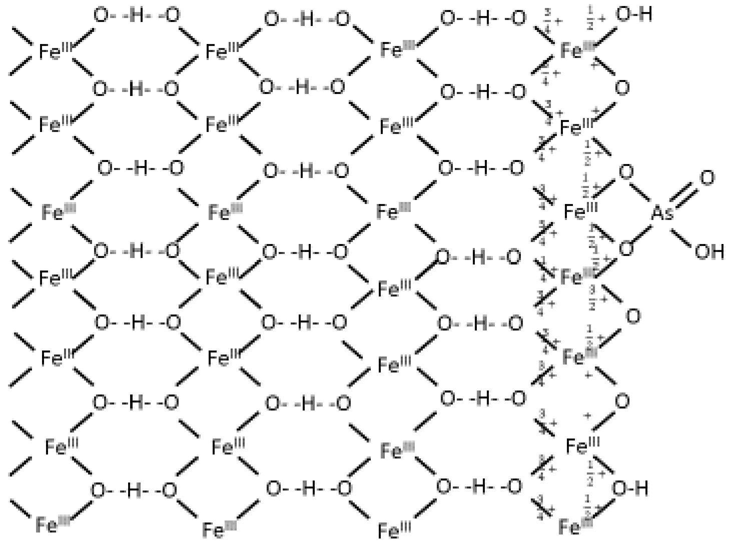

Figure S5. Simplified 2-dimensionalized structure for goethite like ferrioxohydroxides, inclusive of the surface (on the right hand side). (A) Standard structure. The blue box indicates the unit cell FeO(OH). For the iron atoms on the right hand side partial charges are indicated; in the electrostatic bonding model they offer 3/4 positive charge to each neighboring oxygen atom, except for the column on the far right where each iron offers a full positive charge to an oxygen atom to its right and half a positive charge to an OH group to its right. Both of the latter bind another iron ion in the same electrostatic way. O and H are supposed to carry 2− and 1+ charges, respectively, which are not indicated. For simplicity for only two columns of the irons within the crystal the charges are indicated. (B) On the right-hand side the dissociation of a hydroxyl ion has left a positive charge on the resin. (C) Partial reduction of the goethite resulting in a copolymer of goethite and magnetite, indicating that the ratio of ferrous to ferric irons need not be one in two but could also be one in any integer number. The reduction of a ferric iron in the chain would do away with one of the sites for

outer shell binding of arsenate (see E). (D) As (A) but with some iron electric charges in the right-hand columns only, and a proton rearranged. (E) and (F): Two modes of binding of arsenic (mostly anionic arsenate binding to B leading to E and arsenite [although here arsenate is shown instead] binding to D leading to F after double hydrolysis, respectively) onto ferrihydrites ((oxyhydr)oxides such as goethite (α-FeO(OH)). E: The 'outer sphere' (electrostatic) mechanism. F: The inner sphere (covalent bonding) mechanism. Below pH = 9, the goethite carries a positive surface change because of dissociation of some of its hydroxyl groups (OH^-). The arsenate is singly or doubly negatively charged (here we show the singly negatively charged form). To account for the observations in [81] we depict that the binding of arsenate to the ferrihydrite is twofold, i.e., electrostatic ('outer-sphere-type') (E) and 'inner-sphere-type' through a covalent bond after hydrolysis (F). Because of the two covalent bonds between the arsenite/arsenate and the ferrioxyhydroxide, the complex in F should be less sensitive to increased ionic strength than expected for the electrostatic interaction alone. It should still be sensitive to highly alkaline pH however, as is the outer sphere mechanism because of deprotonation of the ferrihydrite. The preferential binding of arsenate to the ferric form will decrease the apparent midpoint potential of the Fe(II)/Fe_2O_3 couple further. This binding will also be prevented by reduction (C) of the positively charged ferric irons in the structure (B).

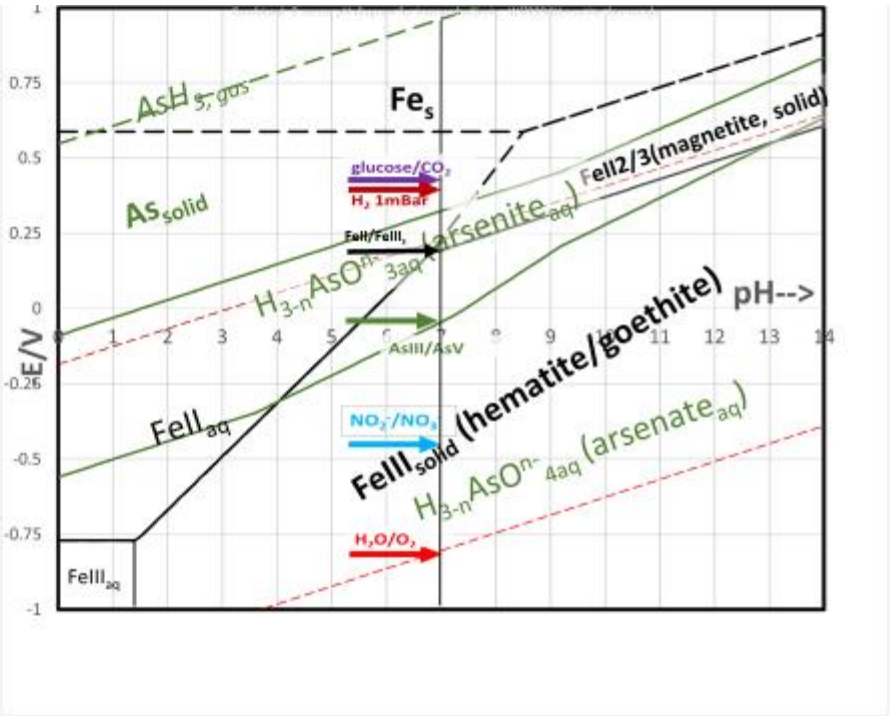

Figure S6. Combined electron potential (negative redox potential −*E*) versus pH diagram for iron and arsenic for groundwater reference conditions, (21% O_2, 0.55 μBar H_2, 10 μM ferrous and ferric iron, 10 nM arsenates and arsenites, 1 mbar arsine. The short vertical black line at the bottom left represents the pKa of the transition between aqueous ferric iron and the solid hematite. The dashed red lines are the borders beyond which protons form hydrogen gas (the upper red line) and water forms molecular oxygen. The bold-face horizontal arrows indicate the midpoint potentials at pH 7 of the indicated redox couples under relevant standard conditions, where the hydrogen partial pressure has been taken to equal 1 mBar. Subscripts gas, s, and aq refer to gaseous, solid and aqueous, respectively.

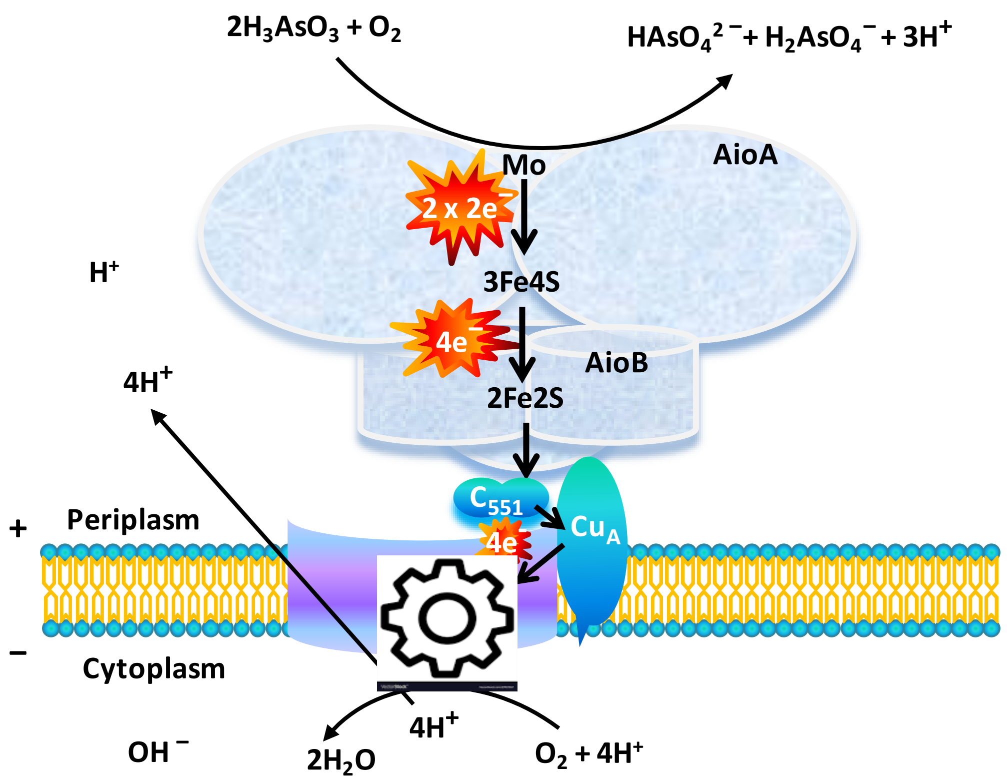

Figure S7. Putative electron transport chain for arsenite oxidation in *Hydrogenophaga* NT14, which can extract Gibbs energy from arsenic oxidation by molecular oxygen. Arsenite in the form of arsenic acid) is oxidized to arsenate at the Molybdenum (Mo) containing active site of the AioA subunit. Subsequently, two electrons are passed to the [3Fe4S] center from where they are then passed one at a time to the [2Fe-2S] centre of the Rieske subunit (AioB). The electrons are next transferred to cytochrome c_{551} bound as a dimer to AioB and from there to the CuA and heme a of the aa_3 cytochrome c oxidase complex. A total of four electrons are required to reduce molecular oxygen to water in the cytoplasm. Coupled to this reduction, four protons are pumped from the cytoplasm into the periplasm, further creating a transmembrane electrochemical potential difference for protons (proton motive force Δp). In addition, the electron pathway involves the translocation of 4 electrons, i.e. 4 negative charges inward across the membrane. The result of the oxidation of 2 extracellular arsenite ions by molecular oxygen therefore is the outward translocation of 8 elementary charges and 4 acid equivalents across the membrane which can subsequently be used to drive the phosphorylation of 2 molecules of ADP to generate ATP if the H^{+-}>/ATP stoichiometry of the H^+-ATPase is 4 [adapted from [120]].

S2. Supplementary text

S2.1. Interaction with sulfur, phosphate, bicarbonate and organic carbon

An exemplary reaction generating the iron oxides is the aerobic oxidation of ferrous disulfide minerals to aqueous ferrous iron and aqueous sulfate:

$$Fe(II)S_2 + {}^7/_2\, O_2 + H_2O \rightarrow Fe^{2+}(aq) + 2\, SO_4^{2-}(aq) + 2\, H^+ \quad \text{(Eq. S1)}$$

This reaction leads to acidification which then stimulates the subsequent oxidation of the ferrous iron to ferric iron by molecular oxygen, as that reaction consumes one proton per iron (Eq. 6). The total reaction becomes:

$$Fe(II)S_2 + {}^{15}/_4\, O_2 + \tfrac{1}{2}\, H_2O \rightarrow Fe^{3+} + 2\, SO_4^{2-} + H^+ \quad \text{(Eq. S2)}$$

The ferric iron formed then precipitates with anions such as arsenate, arsenite, phosphate, or hydroxide (OH^-) coming from water. In the latter case ferric hydrite, goethite or hematite is formed (Eq. 7). The production of protons in the latter reaction makes

the formation of rust from ferrous iron autocatalytic; rust seems to create rust. The same applies to the total reaction:

$$FeS_2 + {}^{15}/_4\ O_2 + {}^{5}/_2\ H_2O \rightarrow FeOOH\downarrow + 2\ SO_4^{2-} + 4\ H^+ \qquad \text{(Eq. S3)}$$

Again, the oxidized iron is not present as ferric ion (Fe^{3+}) but as ferric hydroxide ($Fe(OH)_3$, goethite ($FeOOH$) or hematite (Fe_2O_3), i.e. as ferric oxyhydroxide ($Fe(III)O(OH)$) in various states of hydration and crystal forms.

The outer sphere mechanism (Figure S5E) suggests that by mere competition anions should reduce the sorption of arsenite to ferrihydrite. Indeed, Freitas *et al.* (2014) [92] found that phosphate, bicarbonate and dissolved organic carbon could directly and indirectly hinder arsenic adsorption to ferric hydroxide [296]; ferric hydroxide surfaces are known for their strong affinity for phosphate ions. Likewise silicates are main competitors of arsenate for sorption sites [297-299].

When $H_2PO_4^-$ and HPO_4^{2-} adsorb, the surface charge of the iron hydroxide becomes negatively charged, hindering *in situ* ferric iron oxide hydrolysis from goethite to ferrihydrite ($FeO_2H + H_2O \rightarrow Fe(OH)_3$) and precipitation of $Fe_2(OH)_6$ [300-302], as $FeO^+H_2PO_4^-$ may not be equally eager to precipitate.

Also high bicarbonate concentrations may affect the sorption. Surface sites will be occupied by HCO_3^- binding to goethite ($FeOOH$) [93,300]. Indeed, bicarbonate is an effective competitor with arsenite and arsenate for adsorption onto goethite under field conditions, where the largest effect was seen at the lowest experimental pH (~6.5) [303]. Sulfate affects arsenic adsorption onto solid phases likewise, depending on redox processes and co-precipitation [72,301]. As compared to ferrihydrite, clays and oxides/oxyhydroxides of aluminium and manganese are also important adsorbents of arsenic, especially in iron deficient systems [304].

S2.2. Bangladesh as case in point

In the main text, we discussed the complexity of the many processes that can determine the concentration of arsenite in groundwater used for drinking water. But are these processes actually relevant? Do they ever occur simultaneously? In this section we discuss the example of Bangladesh.

By world standards most of the Bangladeshi groundwaters have high concentrations of both iron (>0.2 mg/L) and arsenic (>10 μg/L), and the arsenic concentration correlates positively with the ferrous iron concentration [186,305]. Bissen and Frimmel (2003) reported more than 60% of the arsenic in Bangladeshi groundwater to be arsenite [306]. Iron oxides are closely associated with the development of highly arsenic groundwaters in Bangladesh. In Bangladesh, highly arsenic groundwaters are characterized by high HCO_3^- concentrations (>0.5 g/l) and low levels of SO_4^{2-} (<1 mg/L) and NO_3^- (<1 mg/L), by high ambient electron potentials -*E* (i.e., by reduced conditions), and by pH values close to or above 7.0 [46]. Bicarbonate adsorption decreased the ability of sediments to adsorb arsenite under conditions similar to those of Bangladesh groundwater [307]. Possibly, the concentration of fine-grained iron oxides in the coarse sediments is one of the many factors simultaneously controlling the development of the groundwater arsenic problem in Bangladesh [308].

We conclude that many of the processes that determine arsenite concentration in groundwater can be relevant simultaneously. They are probably relevant in the case of Bangladesh.

S2.3. Abiotic arsenite management strategies

S2.3.1. Chemical treatment of drinking water

Numerous and diverse types of technologies have been and are being developed for the removal of arsenic contamination from groundwater. Many of these are successful in

the laboratory but less successful in the field. Environmental engineers, scientists, and experts used to focus on the use of chemical oxidants or chemical sorbents for arsenic removal, as these methods are rapid and can be conducted under controlled *exsitu* conditions. Common technologies employ advanced oxidation processes, (electro)coagulation / co-precipitation, lime treatment, adsorption onto sorptive media such as ion-exchange resins, membrane filtration (nano-filtration) and reverse osmosis. The oxidation processes are simple and low-cost but remove only part of the arsenic. The sorption techniques are relatively well defined and commercially available but produce toxic solid waste and require high maintenance operational cost as well as replacement or regeneration. Membrane techniques have high removal efficiency and less toxic solid waste but are costly in terms of operation and maintenance [86].

Full abiotic oxidation of arsenite by atmospheric oxygen takes weeks [77] or years [26]. Complete oxidation by ozone may be achieved in less than 20 minutes [309] Other chemical oxidants like chlorine, hypochlorite and permanganate can also rapidly oxidize arsenite to arsenate under a wide range of conditions [86], according to the following processes:

$$\mathrm{H_3AsO_3 \ + \ HClO \rightarrow HAsO_4^{--} \ + \ Cl^- \ + \ 3H^+} \qquad \text{(Eq. S4)}$$

$$\mathrm{3H_3AsO_3 \ + \ 2KMnO_4 \rightarrow 3HAsO_4^{--} \ + \ 2MnO_2 \ + \ 2K^+ \ + \ 4H^+ \ + \ H_2O} \qquad \text{(Eq. S5)}$$

S2.3.2. The chemical reactions involved in 'Solar oxidation and removal of arsenic' (SORAS).

Much of the ferrous iron (Fe(II)) is oxidized spontaneously to ferric iron by the molecular oxygen in the solution (see also Eq. 6):

$$Fe^{II} + \frac{1}{4}O_2 + H^+ \Rightarrow Fe^{III} + \frac{1}{2}H_2O \qquad \text{(Eq S6)}$$

Most of the ferric iron precipitates as goethite ($(Fe^{III}OOH)_n$; or hematite or some other ferrihydrite; see also Eqs. 7 and 8):

$$n \cdot Fe^{III} + 2 \cdot n \cdot H_2O \Rightarrow (Fe^{III}OOH)_n \downarrow + 3 \cdot n \cdot H^+ \qquad \text{(Eq S7)}$$

Citrate (*'cit'*) from the added lemon juice keeps some ferric iron in solution and subject to solar irradiation, which then activates it:

$$Fe^{III} + citrate \Leftrightarrow Fe^{III}citrate \qquad \text{(Eq. S8)}$$

$$Fe^{III}citrate + h \cdot \nu \Rightarrow Fe^{III*}citrate \qquad \text{(Eq. S9)}$$

The activated ferric iron exchanges an electron with ferrous iron:

$$Fe^{III*} + Fe^{II} \Leftrightarrow Fe^{II*} + Fe^{III} \qquad \text{(Eq. S10)}$$

Photochemical activation of oxygen uses the photo-activated ferrous iron:

$$Fe^{II*} + O_2 \Leftrightarrow Fe^{III} + O_2^{\cdot-} \qquad \text{(Eq. S11)}$$

$$Fe^{II*} + O_2^{\cdot-} + 2H^+ \Rightarrow Fe^{III} + H_2O_2 \Leftrightarrow 2OH^{\cdot} \qquad \text{(Eq. S12)}$$

This is stimulated by the acidity of the lemon juice. Arsenite is then oxidized:

$$H_3AsO_3 + 2OH^{\cdot} \Leftrightarrow H_2AsO_4^- + H^+ + H_2O \qquad \text{(Eq. S13)}$$

The anionic arsenate binds to the goethite, which causes it to precipitate (see also Eq. 3):

$$(Fe^{III}OOH)_n \downarrow + H_2AsO_4^- \Rightarrow (Fe^{III}OOH)_{n-1}Fe^{III}OH_2AsO_4 \downarrow + OH^- \qquad \text{(Eq. S14)}$$

The supernatant will therefore be depleted from iron and arsenic, as further illustrated by Fig. S8.

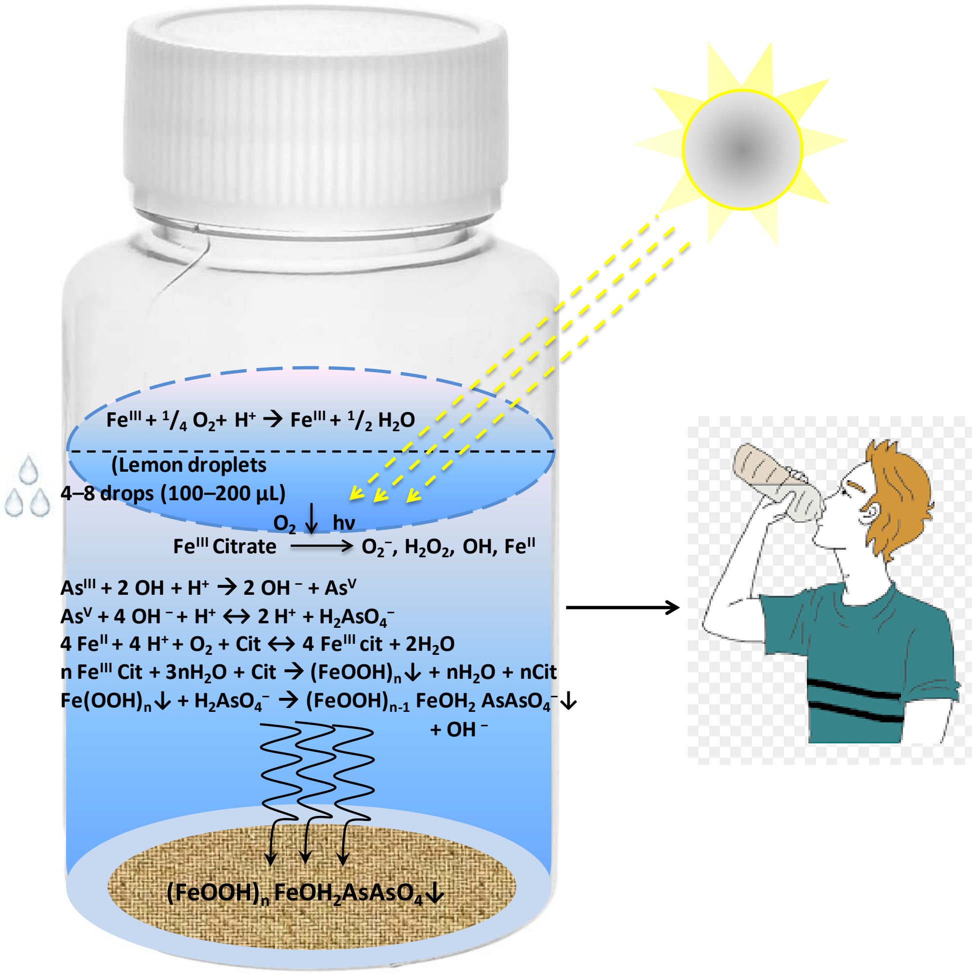

Figure S8. Principle of the solar method with illumination, photochemical formation from O_2 of reactive oxidants for the oxidation of As(III) to As(V) and precipitation of Fe(III) (hydr)oxides with adsorbed As(V).

S2.3.3. Adsorption

Adsorption is one of the most widely used methods to remove contaminants in water treatment plants. Its advantages include relatively high arsenic removal efficiencies [310,311], easy operation, and handling [312], and cost-effectiveness [313]. Maitlo (2019) found that their absorption method could be more than 95% efficient in the remediation of As(III) and As(V). It does not require additional chemicals, and a semi-skilled worker can easily operate the procedure, making it feasible to impoverished communities with unstable electrical supplies [314]. This method depends on a Van der Waals separation process, based on dynamic electric forces between molecules and the sorbent. However, adsorption of arsenic strongly depends on the sorbent concentration and pH. At low pH, arsenate adsorption is favored, whereas for arsenite, maximum adsorption can be obtained between pH 4 and 9 [315]. The effectiveness of adsorption in arsenic removal can be compromised by the type of adsorbent itself. A wide variety of sorbents has already been studied and these include activated carbon, coal, red mud, fly ash, chicken feathers, kaolinite, montmorillonite, goethite, zeolites, activated alumina, titanium dioxide, iron hydroxide, zero-valent iron, chitosan, and anion-exchange resins [26]. Anion exchange resins are commonly used for the treatment of arsenic contamination where the oxy-anionic species of arsenate ($H_2AsO_4^-$, $HAsO_4^{2-}$ and AsO_4^{3-}) are effectively exchanged with the anionic charged functional group of the resin, thus producing effluents with low

concentration of As(V) [316]. Ferric iron can remove arsenite from water either by acting as a sorbent co-precipitant, or by behaving as an oxidant [317]. Arsenic release from soils at high pH may be partly attributed to the mechanism of pH-related anion desorption, that is, replacement of arsenite and arsenate ions bound in the sorption complexes by hydroxide ions (OH^-). Arsenate adsorption on oxides and clays is maximum at low pH and decreases rapidly at neutral or alkaline pH, that is, at pH ranging from 5 to 9, depending on the soil components: at pH 9 for aluminum oxides, pH 7 for iron oxides and pH 5 for clays [318]. Arsenite adsorption is maximal at pH 8–9 [54,319,320]. Arsenic mobilization in strongly acidic conditions is probably caused by dissolution of soil iron oxides. Soil carbonates are responsible for the buffering capacity against acidification [318].

Arsenic release at high pH seems to be the one caused by the dissolution of the soil's organic components, including humic substances [321]. The content of organic matter in the soil appears to be a crucial factor that affects the solubility of arsenic in extremely alkaline conditions. The release of arsenic probably involves a combination of organic matter dissolution, anion desorption and chelation. The hydroxides and oxyhydrates of iron and manganese significantly influence the concentration of trace elements such as arsenic in natural waters, because such trace elements adsorb onto their surfaces [322]. As discussed above (Figure S5), the surface of iron oxyhydrates is positively charged and provides an attractive surface to arsenic containing oxy-anions. Because the oxyhydrate of manganese is negatively charged, arsenic will only be adsorbed when the surface is positively charged by additional Mn^{2+} ions. Manganese is usually present in groundwater as a divalent ion (Mn^{2+}) and is considered a pollutant mainly because of its organoleptic properties [323]. In general, manganese dioxide can readily oxidize arsenite to arsenate by ($H_3AsO_3 + MnO_2 \Leftrightarrow HAsO_4^{2-} + Mn^{2+} + H_2O$); the released Mn^{2+} ions adsorb onto the manganese dioxide, giving it a positive surface charge and leading to an enhancement in the removal (through adsorption) of arsenate present initially or produced as a result of the arsenite oxidation [324]. Such (oxide/hydroxide surface) interactions between iron, manganese, and arsenic are important for adsorption processes in natural waters.

Another adsorbent was prepared by *in situ* precipitation of $Fe(OH)_3$ on the surface of activated Al_2O_3. This adsorbent was used in a complex process involving ion exchange and adsorption to remove ammonium ions, humic substances, and arsenic from drinking water [325]. A great advantage of this type of adsorbent is that it can be used in adsorption columns [326]. More recently, graphene oxide (GO) and its composites have been found to be adsorbents. They might be promising for the adsorption of various water pollutants due to their unique physicochemical characteristics [327].

S2.3.4. Coagulation, precipitation, and filtration

Some common coagulants used in water treatment are also effective in removing arsenic from groundwater. Titanium(IV) Sulfate [$Ti(SO_4)_2$] can enhance As(III) removal. The removal efficiency of As(III) was 90% at a coagulant dose of 25 mg/L [328]. Other coagulants include halotrichite (also known as feather alum, which is a highly hydrated sulfate of aluminum and iron (i.e., $FeAl_2(SO_4)_4{\cdot}22\,H_2O$)), activated alumina ($Al_2O_3$), ferric chloride and ferric sulfate. Ferric salts have been shown to be more effective in removing arsenic than alum over a wider range of pH. In both cases, pentavalent arsenic is removed more effectively than trivalent arsenic [19,77]. Membrane filtration is used to clean arsenic and other contaminants from drinking water throughout the world. Several studies reported that arsenic can also be removed from groundwater by a nano-filtration (NF) membrane configuration [329,330]. Galambos (2006) showed that nano-filtration (NF), combined with $KMnO_4$, constitutes an efficient method for producing water that can meet drinking water standards. When using nano-filtration, residual wastewater was generated that was very high in arsenic and required further treatment. The arsenic concentration in this residue was 0.5–0.7 mg/L, achieved by reverse osmosis membrane treatment followed by precipitation of the arsenic content with H_2S [330]. In this study, the retentate of the nano-filtration of well-water was used as feed solution and $KMnO_4$ was used as oxidizing agent

with lime softening (pure $Ca(OH)_2$ and commercial slaked lime). According to the report, from the concentrate arsenic was precipitated with hydrogen sulfide, which had been produced from iron sulfide with hydrochloric acid. The precipitate was removed by microfiltration and sedimentation.

Arsenic can also be precipitated in the form of As_2S_3 by adding Na_2S [331]. Sulfide reactions with metal ions such as Cu^{2+}, Cd^{2+}, and Fe^{2+} and H_3AsO_3, occur via the addition of a sulfide agent (Na_2S solution) to the arsenic-containing wastewater:

$$Na_2S + H_2SO_4 \rightarrow H_2S + Na_2SO_4 \quad \text{(Eq. S15)}$$

$$3H_2S + 2H_3AsO_3 \rightarrow As_2S_3 \downarrow + 6H_2O \quad \text{(Eq. S16)}$$

In these precipitation strategies, high amounts of hazardous arsenic-concentrated secondary sludge are produced however. The management of this sludge is necessary so as to prevent secondary pollution of the environment. Treatment of sludge is costly. These limitations make this process less feasible, especially in field conditions. Moreover, these technologies are not sustainable or affordable to most local communities due to initial cost and high maintenance requirements and are therefore not extensively used.

S2.3.5. Managed Aquifer Recharge

Some conventional methods do not require any chemicals. Managed aquifer recharge (MAR) is based on the principle of infiltrating surplus (wet season: monsoon) fresh water into a shallow aquifer for storage, followed by withdrawal during periods of water deficit (dry season) [332,333]. At the MAR sites in Bangladesh, fresh surface water was collected from ponds and from harvested rooftop rainwater, and stored in confined aquifers using relatively large diameter infiltration wells [334,335]. In order to reduce the salinity, turbidity, and microbial contamination, a sand filter was used to treat the pond water (termed "infiltration water") before injection into the aquifer. MAR is a promising technology for providing arsenic and saline free water especially in the coastal belt of Bangladesh [336,337]. MAR systems take advantage of natural water purification processes, *e.g.*, filtration, sorption, and biodegradation during sediment-water interactions in the aquifers [338,339]. When water is stored in an anoxic aquifer, dissolved oxygen (DO) from the infiltrated surface water oxidizes Fe(II) in native groundwater and converts it into Fe-hydroxides, which are adsorbents for arsenate, arsenite and Mn (see above and [88,340]). A risk of MAR has been noted however: the direct injection of oxic water into the subsurface may also enhance arsenic release into groundwater by the dissolution of sulfide minerals naturally present in the subsurface [96,97]. It has been claimed that As and Mn can also adsorb onto sedimentary organic matter and clay minerals in addition to the targeted Fe-hydroxides [341].

Due to reductive dissolution of the Fe-hydroxides, changes in Fe and As concentrations have been observed at several MAR systems, and the levels of Fe and As have occasionally exceeded the drinking water regulations [342]. Other a-chemical remediation strategies include the use of dug wells, household filters, rainwater harvesting, deep tube wells, and the construction of village piped water supplies. Success varies, largely depending on local resources and geological conditions [322].

S2.4. Sulfur

Apart from iron- and arsenic-cycling microorganisms, also microorganisms performing other redox reactions, such as sulfur-cycling, may impact arsenic and iron mobilization and immobilization [115]. Sulfur-oxidizing bacteria can grow aerobically with free or arsenic-bound sulfur as redox substrate, thereby producing sulfite or sulfate and transforming thioarsenates to arsenate [343]. Sulfide, which is produced by sulfur- and sulfite-reducers and has an apparent midpoint electron potential relative to sulfur (negative midpoint potential -E_0') of 0.28 V [71]; 0.097 according to [55], but there is some unclarity in the relevant Table in reference [55]), can thereby reduce arsenate (-E_0'= -0.05 V; Figure

S2, Figure S6) better than ferric iron (-E_0′= 0.20 V; Figure S6; ferric iron in hermatite). Sulfite (-$E_0{}'$ = 0.53 V [71, 55] relative to sulfate) can do the same and even reduce ferric iron to hematite, thereby increasing the sulfate levels and precipitating arsenate. High sulfate concentrations are generally associated with low arsenic [344]. Sulfide can also immobilize arsenic by the co-precipitation of arsenic and iron sulfide minerals [115,345]. Several studies suggested that arsenic-thio compounds comprised ≥ 50% of the total arsenic in sulfidic environments [346-348]: sulfide and arsenite can engage in strong interactions, which may overcome redox potential limitations of Figure 2. The sulfide may thereby reduce two arsenites to elemental arsenic, the resulting sulfur being added to three other arsenites to produce thioarsenate in a Gibbs energy lowering reaction. Indeed, abiotic co-incubation at pH = 7 led to virtually instantaneous formation of thioarsenate [349]:

$$5\ H_3As^{III}O_3 + 3\ H_2S^{-II} \Rightarrow 2\ As^0\downarrow + 3\ H_3As^{III}O_3S^0 + 6\ H_2O \qquad \text{(Eq. S17)}$$

The elemental arsenic precipitates in black flocks. Intramolecular electron transfer then leads to thioarsenate, which may hydrolyze to arsenate and hydrogen sulfide:

$$H_3As^{III}O_3S^0 + H_2O \Rightarrow H_3As^{V}O_3S^{-II} + H_2O \Leftrightarrow H_3As^{V}O_4 + H_2S^{-II} \qquad \text{(Eq. S18)}$$

The net result then is the disproportionation of arsenite to elemental arsenic and arsenate:

$$5\ H_3As^{III}O_3 \Rightarrow 2\ As^0 + 3\ H_2O + 3\ H_3As^{V}O_4 \qquad \text{(Eq. S19)}$$

In addition dithioarsenate, trithioarsenates and tetrathioarsenates are formed [349] by the exchange of oxygen and sulfur di-anions:

$$H_3As^{V}O_3S^{-II} + 3\ H_2S \Leftrightarrow H_3As^{V}O_2S^0S^{-II} + H_2O + 2\ H_2S \Leftrightarrow$$

$$H_3As^{V}OS^0S^0S^{-II} + 2H_2O + H_2S \Leftrightarrow H_3As^{V}S_3S^{-II} + 3H_2O \qquad \text{(Eq. S20)}$$

Edwardson et al (2014) reported trithioarsenite, which is extremely reactive and oxidizes to tri- and tetra-thioarsenate. Thioarsenite oxidation can be induced by both oxygen and elemental sulfur [350]. Tri- and tetra-thioarsenate are both unstable and eventually decompose to a mixture of arsenite, arsenate, and di- and monothioarsenate [351].

Sulfur-oxidizing bacteria can grow with free or arsenic-bound sulfur as substrate, transforming indirectly or directly arsenite and thioarsenates to arsenate [343]. Sulfide (-$E_0{}'$= 0.28 V versus elemental sulfur) produced by sulfur- and sulfate-reducers can reduce aqueous ferric iron (-$E_0{}'$= -0.77 V; but it is 0.2 V for ferric iron in hematite, so that the reduction will be limited) and arsenate (-$E_0{}'$= -0.06 V), and also immobilize arsenic by the precipitation of arsenite sulfide (As_2S_3) and iron sulfide minerals [115,345]. Whether or not the sulfide leads to much net arsenite immobilization when the ferrihydrite also gets reduced is complex and unclear [115], understandably when it is considered that the aqueous ferric iron concentration is very low and the electron potential of the hematite lies close to that of the sulfide/sulfur couple; see above). When there is sufficient oxygen the sulfur can be oxidized to sulfite or sulfate. High sulfate concentrations tend to be associated with low arsenic [344]. SAR cycling was accompanied by an increase in sulfate (which did not occur in the reference well) [238], probably evidencing the *activity* of sulfide or sulfite oxidizing bacteria. Paradoxically more of the sulfur oxidizing genus *Sulfuricurvum* seemed to be present in the reference well than in the SAR well, perhaps again because they are microaerobes and thereby compromised by periodic high oxygen levels. A previous study has already explained why this type of freshwater ecosystem contained the highest amount of bacteria belonging to the anaerobic-to-microaerobic sulfur-oxidizing genus *Sulfuricurvum* [352]. Other studies reported that *Sulfuricurvum* can be a facultative anaerobe, chemolithoautotrophic and is metabolically versatile. In anaerobic and microaerophilic environments, sulfur (S)-oxidizing bacteria can contribute to pyritic-S dissolution by the oxidation of solid sulfur such as sulfide, elemental S and thiosulfate in arsenic rich subsurface groundwater aquifers and sediments [254,258,352-355].

S2.5. Microorganisms using electron acceptors other than molecular oxygen

Anaerobic denitrifying ferrous iron and arsenite oxidizers could be an alternative to aerobic iron and arsenite oxidizers, and a promising option for bulk arsenic removal because this would not require oxygen injection. Such microorganisms, i.e. *Dechloromonas*, *Acidovorax* and *Paracoccus,* were found in the SAR wells [238]. *Acidovorax* and *Paracoccus* are known as facultative anaerobes [356,357]. Similar organisms had been identified in the earlier cultivation-dependent and cultivation-independent analyses of the same wells [107,108,235]. Aerated tank water could provide molecular oxygen as an electron acceptor to ammonia oxidizing bacteria, causing nitrate to be produced biologically. Nitrate was indeed observed in the SAR rooftop tank (Figure 4) after 10 cycles of operation, and neither in the SAR well initially, nor in the reference well [238]. The nitrate injected into the aquifer with the aerated water from the tank may have served as electron acceptor to anaerobic denitrifying arsenite and iron oxidizers once the oxygen had ran out. Harvey et al. (2002) showed that injection of nitrate into a subsurface aquifer in Bangladesh resulted in the immobilization of arsenic [94]. The aerobic biological conversion of ammonia to nitrate and subsequent anaerobic reduction of the nitrate by microbes using arsenite or ferrous iron as electron donor might provide for a cheap technology for the *in situ* or *ex situ* removal of arsenic from drinking water; an alternative to oxygen-based SAR.

S2.6. Methylation and methanogenic bacteria

Arsenic is also available in organic forms, *i.e.,* in conjunction with carbon [46]. The methylated organic forms are mostly observed in marine water systems while the inorganic forms (arsenite and arsenate) are more abundant in freshwater [358]. Inorganic arsenic compounds can be methylated (hydroxyl groups being replaced by methyl groups) by microorganisms to from organic arsenic compounds, *e.g.,* MMA and DMA [359,360] (Figure 8). Under anaerobic conditions, methylated arsenic can be further transformed by microorganisms into volatile forms such as arsine [AsH_3] and mono-, di- and tri-methyl arsine [H_2AsCH_3; $HAs(CH_3)_2$; $As(CH_3)_3$]. Biomethylation contributes significantly to aquifer arsenic cycling and may serve as a link between surface and subsurface by transforming inorganic arsenic to organic arsenic and *vice versa* [251] (see also Figure 8)

In methanogenic bacteria, methylation of inorganic arsenic under anaerobic conditions is coupled to the methane biosynthetic pathway, the methyl group being added to the arsenic rather than to hydrogen [165]. The process proceeds by reduction of arsenate to arsenite followed by methylation to dimethylarsine. As methyl donor SAM (S-Adenosyl methionine) is a required coenzyme for arsenic methylation. Arsenate is reduced to arsenite in a reaction thought to depend on GSH (glutathione). Arsenite methyltransferase (AS3MT also known as CYT19) catalyzes the oxidative methylation of arsenite using SAM as the methyl donor, forming monomethylarsonic acid (MMA(V)) and SAH (S-Adenosylhomocysteine). MMA(V) is reduced to monomethylarsonous acid (MMA(III)) before a subsequent oxidative methylation step yielding dimethylarsenic acid (DMA(V)) and SAH. Little is known regarding *in vivo* reduction of DMA(V) to DMA(III) by MMA(V) reductase also known as GST-Ω (Glutathione S-transferase). Enzymes capable of catalyzing the reactions illustrated by Figure 8 include Cyt19 [361], arsenite methyltransferase and methylarsonite methyltransferase (two activities of one enzyme) [362], and MMA(V) reductase (also known as GST-Ω) [363].

S2.7. Arsenite oxidase structure and molecular function

Arsenite oxidase has been purified from the heterotrophic bacterium *Alcaligenes faecalis* and fully characterized. Its crystal structure has two heterologous subunits. The large catalytic (α-) subunit (AioA) contains molybdenum bound to two pterin cofactors and a 3Fe-4S cluster. It is structurally related to members of the dimethyl sulfoxide (DMSO) reductase family of molybdo-enzymes. The small (β-) subunit (AioB) presumably functions as an electron shuttle (Figure S7) and has a Rieske-type [2Fe-2S] cluster [221,364]. The

arsenite oxidase operon usually contains four genes: *aioA, aioB, cytC* and *moeoA1* (which encodes an enzyme involved in molybdenum cofactor biosynthesis), which are transcribed when the organism is grown in the presence of arsenite.

Amicyanin or periplasmic cytochrome *c* can act as an electron acceptor for the arsenite oxidase [120,365]. The cytochrome may then shuttle the electrons to the cytochrome c oxidase aa_3 complex, where they are transferred across the membrane and reduce intracellular molecular oxygen under uptake of intracellular protons. Oxygen and water freely diffuse across the membrane. At an extracellular pH around 7 the overall reaction is:

$$2\ H_3AsO_3 + O_2 + 8\ H^+_{cytosolic} \rightarrow HAsO_4^{2-} + H_2AsO_4^- + 11\ H^+_{periplasmic} \qquad \text{(Eq. S21)}$$

As compared to the a-biotic extracellular oxidation of arsenite, *i.e.,*

$$2\ H_3AsO_3 + O_2 \rightarrow HAsO_4^{2-} + H_2AsO_4^-, + 3\ H^+_{periplasmic} \qquad \text{(Eq. S22)}$$

this corresponds to the outward pumping of 8 protons, *i.e.,* 2 protons per electron stemming from arsenic:

$$8\ H^+_{cytosolic} \rightarrow 8\ H^+_{periplasmic} \qquad \text{(Eq. S23)}$$

Four of these protons are chemically coupled to the reduction of O_2 to water, whilst the other 4 are due to proton pumping *sensu strictu* [366]. The midpoint *potential* difference between the arsenic redox couple and the O_2/H_2O couple is 760 mV (Fig. S6). At a transmembrane *electric potential* of 180 mV and based on the midpoint potentials, this provides for a thermodynamic efficiency of almost 50% (760/(2*180)). Such thermodynamic (in)efficiencies are not uncommon, and their shortfall of 100 % serves to accelerate biochemical network processes [367].

S3. Nomenclature

Bacteria that use organic compounds as their source of carbon and Gibbs energy are called heterotrophic; those that use inorganic carbon compounds such as carbon dioxide autotrophic; those that use inorganic material as their Gibbs energy source chemolithotrophic. Bacteria that use oxygen as their electron acceptor are aerobic; those that use a compound other than oxygen (e.g., nitrate or sulfate) are anaerobic; and those that can utilize both oxygen and other compounds as TEAs are facultative aerobes.

S4. Abbreviations

α-, anhydrous; aq, aqueous; Aio/Arx, various arsenite oxidase types; Arr, dissimilatory arsenate reductase; Ars(A)B, arsenite efflux pump; ArsC, detoxifying arsenate reductase; As, arsenic; As(III), arsenite; As(V), arsenate; aSAR, abiotic SAR; bSAR, biotic SAR; CAO, chemolithoautotrophic arsenic oxidizers; CFU, colony forming unit; DMA, $C_2H_6AsO_2$ dimethylarsine; DMAA, dimethylarsinic acid; DOM, dissolved organic matter; *E*, redox potential; E_0' standard transformed redox potential at the relevant pH [55]; FBA, flux balance analysis; Fe(III), ferrous iron; Fe(III) ferric iron; GlpF, aquaglyceroporin GlpF; GSH; reduced glutathione or microbial equivalent; GSSG; oxidized glutathione; HAO, heterotrophic arsenic oxidizers ; MMA, monomethylarsonic acid, CH_5AsO_3; OTU, operational taxonomic unit = microbial quasi species; PCR, polynucleotide chain reaction; Pit, phosphate (PO_4^{3-}) inorganic transport system; Pst, phosphate specific transport system; rRNA, ribosomal RNA; s, solid; SAR, Subsurface Arsenic Removal technology; SIR, Subsurface Iron Removal technology; TMAO, trimethylarsine oxide; TEA; terminal electron acceptor, WHO, World Health Organization; Y, growth yield.
